# Therapeutic Impact of Vericiguat on Ventricular Remodeling in a Pressure-Overload Heart Failure Model

**DOI:** 10.3390/life15111763

**Published:** 2025-11-18

**Authors:** Wen-Rui Hao, Chun-Chao Chen, Fu-An Li, Huan-Yuan Chen, Ju-Chi Liu, Tzu-Hurng Cheng, Jin-Jer Chen

**Affiliations:** 1 Division of Cardiology, Department of Internal Medicine, Shuang Ho Hospital, Ministry of Health and Welfare, Taipei Medical University, New Taipei City 23561, Taiwan; b8501043@tmu.edu.tw (W.-R.H.); b101092035@tmu.edu.tw (C.-C.C.);; 2 Institute of Biomedical Sciences, Academia Sinica, Taipei City 115201, Taiwanjc8510@yahoo.com (J.-J.C.); 3 Department of Biochemistry, School of Medicine, College of Medicine, China Medical University, Taichung City 404328, Taiwan; 4 Division of Cardiology, Department of Internal Medicine, Graduate Institute of Clinical Medical Science, China Medical University Hospital, Taichung City 404327, Taiwan

**Keywords:** pressure-overload heart failure, transverse aortic constriction (TAC), ventricular remodeling, Vericiguat, soluble guanylate cyclase (sGC) stimulation, cardiac hypertrophy, myocardial fibrosis, proteomic analysis

## Abstract

Pressure-overload-induced heart failure is characterized by pathological ventricular remodeling, including hypertrophy and fibrosis, which compromise cardiac function and worsen outcomes. Vericiguat, a soluble guanylate cyclase (sGC) stimulator, has shown therapeutic promise in heart failure with reduced ejection fraction (HFrEF). This study evaluated its antihypertrophic, antifibrotic, and metabolic effects in a murine pressure-overload model. Male C57BL/6 mice (~25 g) underwent transverse aortic constriction (TAC) and received oral Vericiguat (10 mg/kg/day) for 14 days. Cardiac hypertrophy was assessed by gross morphology and heart weight; fibrosis was quantified using Masson’s trichrome and Picrosirius red staining. Collagen deposition and wall stress indices were measured by image analysis. Proteomic profiling of fibroblast- and myocyte-enriched tissues identified differentially expressed proteins (DEPs) across metabolic, structural, mitochondrial, and signaling pathways. Vericiguat significantly reduced heart weight and attenuated TAC-induced hypertrophy. Histological staining revealed marked reductions in myocardial fibrosis and collagen accumulation in the Vericiguat-treated TAC group compared to untreated TAC controls. Quantitative analysis demonstrated improved wall stress indices. Proteomic data showed consistent modulation of DEPs, with restoration of mitochondrial and energy-regulating proteins suppressed by TAC, indicating enhanced bioenergetic support. Collectively, Vericiguat mitigates pressure-overload-induced remodeling through coordinated antihypertrophic, antifibrotic, and metabolic reprogramming mechanisms. These findings support its potential as a therapeutic strategy for heart failure and warrant further clinical investigation.

## 1. Introduction

Heart failure is a clinical syndrome in which the heart cannot pump enough blood to meet the metabolic demands of peripheral tissues under normal filling pressures. It is broadly classified into two types: heart failure with preserved ejection fraction (HFpEF) marked by impaired relaxation and diastolic dysfunction, and heart failure with reduced ejection fraction (HFrEF) defined by impaired systolic contractility and progressive ventricular dilation. HFrEF remains a major cause of morbidity and mortality worldwide. Standard pharmacological therapies—including angiotensin-converting enzyme (ACE) inhibitors, angiotensin receptor blockers, beta-blockers, mineralocorticoid receptor antagonists, and sodium–glucose cotransporter 2 inhibitors—have improved survival and symptom control. However, these agents primarily target neurohormonal pathways and often fail to reverse structural remodeling, particularly myocardial fibrosis and chamber dilation, which drive disease progression [1]. The pressure-overload heart failure model, typically induced by transverse aortic constriction (TAC), reproduces key features of hypertensive and valvular stress, including increased wall tension, collagen deposition, and maladaptive remodeling. Despite guideline-directed therapy, many patients continue to experience recurrent hospitalizations and progressive dysfunction, underscoring the need for novel interventions that directly address underlying pathophysiology [2].

Soluble guanylate cyclase (sGC) stimulators are a distinct class of therapeutics that enhance cyclic guanosine monophosphate (cGMP) signaling independently of nitric oxide. This pathway regulates vascular tone, mitochondrial function, and antifibrotic signaling. Vericiguat, the first sGC stimulator approved for HFrEF, has demonstrated clinical efficacy in reducing cardiovascular death and heart failure-related hospitalizations, particularly in patients with recent decompensation [3,4,5]. The VICTORIA trial confirmed its benefit in high-risk populations, while the VICTOR study further validated its safety and effectiveness across diverse subgroups, including patients with renal impairment or diabetes [6]. Beyond symptomatic relief, Vericiguat has shown potential in preclinical models to mitigate structural remodeling. In pressure-overload and ischemic injury settings, it attenuated myocardial fibrosis, preserved mitochondrial integrity, and reduced inflammatory signaling through modulation of AMPK/Nrf2/NLRP3 and TGF-β1/Smad2/3 pathways [7,8,9]. These findings suggest that Vericiguat may exert direct antifibrotic effects by modulating fibroblast activity and extracellular matrix remodeling, complementing its role in functional improvement. Collectively, clinical and experimental evidence supports Vericiguat as a promising adjunctive therapy in HFrEF, capable of addressing both symptomatic burden and structural deterioration. Its unique mechanism of action positions it as a valuable addition to the evolving heart failure treatment paradigm.

Although Vericiguat has demonstrated clinical efficacy in reducing cardiovascular events among patients with HFrEF, its cellular and histological effects—particularly under pressure-overload conditions—remain insufficiently characterized. Most clinical trials, including VICTORIA and VICTOR, have prioritized symptomatic relief and event reduction, while its impact on myocardial architecture and fibrotic remodeling has received limited attention [3,4]. Emerging preclinical studies suggest that Vericiguat may exert anti-fibrotic effects through diverse molecular pathways, though these mechanisms are not fully delineated. For example, modulation of the AMPK/Nrf2/NLRP3 axis has been shown to reduce pyroptosis and inflammatory signaling in coronary microembolization models [7], while suppression of TGF-β1/Smad2/3 signaling has been associated with decreased collagen deposition in atrial tissue [8]. However, whether these pathways are similarly engaged in pressure-overload-induced ventricular remodeling remains unclear. Additionally, the potential interaction between Vericiguat and CaMKII-related signaling, implicated in arrhythmogenesis and maladaptive remodeling, warrants further investigation. Although some studies propose that Vericiguat may stabilize intracellular calcium dynamics and reduce oxidative stress, direct evidence in pressure-overload contexts is lacking [1].

To address these gaps, this study examined the therapeutic effects of Vericiguat in a TAC-induced mouse model, which replicates key features of hypertensive and valvular heart disease, including elevated wall stress, collagen accumulation, and progressive ventricular dysfunction [5]. Vericiguat was administered post-surgery to assess its ability to mitigate fibrotic remodeling and restore myocardial architecture. Histological evaluation used Masson’s trichrome staining to visualize fibrotic tissue and Picrosirius red staining under polarized light to quantify collagen deposition. These methods provide reliable measures of extracellular matrix expansion, a hallmark of pathological remodeling. In parallel, functional indices such as wall stress parameters were analyzed to link structural changes with biomechanical outcomes. To further define Vericiguat’s mechanistic profile, proteomic analysis and cell-type-specific profiling of fibroblasts and myocytes were performed. This approach enabled identification of differentially expressed proteins (DEPs) associated with extracellular matrix turnover, mitochondrial integrity, and inflammatory signaling. By integrating histological, functional, and proteomic endpoints, this study clarifies Vericiguat’s role in structural recovery and provides mechanistic insights relevant to its clinical application.

## 2. Materials and Methods

### 2.1. Materials

Vericiguat (BAY 1021189), a sGC stimulator, was obtained from Bayer AG (Leverkusen, Germany) and prepared in sterile saline for in vivo administration. The dosing regimen was adapted from prior studies demonstrating efficacy in rodent models of cardiac injury and remodeling [7,10]. Masson’s trichrome and Picrosirius red staining kits (Sigma-Aldrich, St. Louis, MO, USA) were used to assess myocardial fibrosis, following protocols validated in pressure-overload models [8,11]. Proteomic sample preparation employed reagents from Thermo Fisher Scientific (Waltham, MA, USA), including lysis buffers, trypsin digestion kits, and tandem mass tag (TMT) labeling reagents. LC-MS/MS analysis was conducted on a Q Exactive Plus Orbitrap system (Thermo Fisher Scientific, Waltham, MA, USA), consistent with established cardiac proteomics workflows [9]. All chemicals and reagents were of analytical grade and used according to manufacturer instructions. Experimental protocols complied with institutional animal research guidelines and were approved by the relevant ethics committee.

### 2.2. Animal Model and Experimental Design

Male C57BL/6 mice (8–10 weeks old, ~25 g) were used for all experimental procedures. Animals were housed under standard laboratory conditions with a 12-h light/dark cycle, controlled temperature and humidity, and free access to food and water. All protocols were approved by the Institutional Animal Care and Use Committee of Academia Sinica (AS IACUC) and complied with ARRIVE guidelines and national regulations on laboratory animal welfare. To investigate pressure-overload–induced cardiac remodeling, mice were randomly assigned to four groups (*n* = 6 per group): (1) wild-type (WT); (2) sham-operated (Sham), undergoing thoracotomy without aortic constriction; (3) transverse aortic constriction (TAC), to induce left ventricular pressure overload; (4) TAC + Vericiguat (Veri), receiving TAC surgery followed by daily oral administration of Vericiguat (10 mg/kg/day) for 14 days, in accordance with established protocols [10,11]. TAC surgery was performed under isoflurane anesthesia, with a 27-gauge needle used to ensure consistent constriction between the innominate and left common carotid arteries. Postoperative care included analgesia and routine monitoring to minimize discomfort. Body weight and overall health were assessed regularly throughout the study.

### 2.3. Induction of Pressure-Overload Heart Failure

Pressure-overload heart failure was induced using the TAC procedure, a well-established model of hypertensive and valvular cardiac stress. Mice were anesthetized with 2% isoflurane in oxygen and placed supine on a heated surgical platform. After a midline cervical incision, the transverse aorta was exposed between the innominate and left common carotid arteries. A 27-gauge needle was positioned alongside the vessel, and a 7-0 silk suture was tied securely around both. The needle was then removed, leaving a reproducible constriction to elevate left ventricular afterload, as previously described [11]. Successful induction of a pressure gradient was confirmed 24 h post-surgery by Doppler echocardiography, measuring peak flow velocity across the constriction site. Mice with inadequate gradients or surgical complications were excluded. Postoperative care included buprenorphine (0.05 mg/kg, subcutaneously) every 12 h for 48 h and daily monitoring for distress, weight loss, or impaired mobility. Animals were housed individually during recovery and returned to group housing once stable. All procedures complied with institutional animal care guidelines and were approved by the relevant ethics committee.

### 2.4. Drug Administration

Vericiguat was administered to mice in the Veri group to evaluate its effects on pressure-overload–induced cardiac remodeling. The compound was freshly prepared in sterile saline before use. Following dosing protocols validated in prior preclinical studies of cardiac injury and remodeling [11], Vericiguat was delivered by oral gavage at 10 mg/kg/day. Treatment began 24 h after TAC surgery and continued for 14 days. This regimen was chosen to capture early remodeling responses and to align with reports of Vericiguat’s efficacy in reducing fibrosis, oxidative stress, and mitochondrial dysfunction in rodent models [7,9]. Drug administrations were performed at the same time each day to minimize circadian variability. Mice were monitored daily for distress, body weight changes, and treatment tolerance, with no adverse effects observed during the study period.

### 2.5. Histological Analysis

Following euthanasia, hearts were excised, rinsed in cold phosphate-buffered saline, and fixed in 10% neutral-buffered formalin for 24 h. Tissues were dehydrated in graded ethanol, cleared in xylene, and embedded in paraffin. Serial transverse sections (5 μm) from the mid-ventricular region were prepared using a rotary microtome and mounted on glass slides for staining. Myocardial fibrosis was assessed with Masson’s trichrome, which highlights collagen fibers in blue and muscle tissue in red. For specific quantification of collagen, Picrosirius red staining was performed and visualized under polarized light microscopy, enabling enhanced detection of birefringent collagen fibers. These complementary approaches have been validated in pressure-overload models [11]. Alongside histological evaluation, heart weight was normalized to both body weight and tibia length to account for post-surgical fluctuations in body mass. This dual normalization strategy enhances the accuracy and reliability of cardiac hypertrophy assessment. Images were acquired using a Nikon Eclipse microscope with a digital camera and analyzed in ImageJ software (version 1.53t; NIH, Bethesda, MD, USA). Fibrotic area was quantified as the percentage of collagen-stained tissue relative to total myocardial area. At least five fields per section and three sections per heart were evaluated to ensure representative sampling. All analyses were performed by investigators blinded to group allocation.

### 2.6. Functional Assessment

Cardiac function was assessed using a Vevo 2100 high-frequency ultrasound system (VisualSonics, Toronto, ON, Canada) equipped with a high-frequency transducer. Mice were lightly anesthetized with 1–1.5% isoflurane to minimize hemodynamic interference. Parasternal long-axis and short-axis views were acquired to evaluate left ventricular (LV) geometry and contractility indices, including LV end-diastolic diameter (LVEDD), end-systolic diameter (LVESD), fractional shortening (FS), and ejection fraction (EF), as previously described [11]. Dynamic wall stress (DWS) was calculated from echocardiographic parameters using the Laplace relationship:

$$DWS=\frac{P\cdot r}{2h}$$

where *P* represents LV pressure, *r* the chamber radius, and *h* the wall thickness. This provides a quantitative measure of biomechanical load and remodeling under pressure overload. For pressure–volume (PV) loop analysis, a 1.2F catheter (Scisense, London, ON, Canada) was advanced into the left ventricle via the right carotid artery under anesthesia (Figure S1). Real-time pressure and volume signals were acquired using LabChart software (ADInstruments, Colorado Springs, CO, USA). Derived parameters included the end-systolic pressure–volume relationship (ESPVR), preload recruitable stroke work (PRSW), and arterial elastance (Ea), which reflect intrinsic contractility and afterload [9,10]. All functional data were normalized to body weight and heart rate to minimize inter-animal variability.

### 2.7. Proteomic Profiling

Cardiac tissue was harvested 14 days after surgery and processed for proteomic analysis. Fibroblast- and myocyte-enriched fractions were isolated by enzymatic digestion and differential centrifugation, following protocols adapted from previous studies [10]. Enrichment was confirmed by marker-based validation: vimentin expression verified fibroblast fractions, while troponin I identified myocyte fractions. This step ensured transparency and reproducibility of the cell-type-specific proteomic workflow. Proteins were extracted in RIPA buffer containing protease and phosphatase inhibitors and quantified using a bicinchoninic acid assay. Equal amounts of protein from each sample were digested with trypsin and labeled with tandem mass tags (TMTs) for multiplexed quantification. Labeled samples were pooled and analyzed by high-resolution liquid chromatography–tandem mass spectrometry (LC-MS/MS) using a Q Exactive Plus Orbitrap system, as previously described [9]. Spectral data were processed with Proteome Discoverer software (version 2.4; Thermo Fisher Scientific, Waltham, MA, USA) and searched against the UniProt mouse database. DEPs were identified using fold-change thresholds and adjusted *p*-values with Benjamini–Hochberg correction. Bioinformatics analysis was performed with DAVID (version 6.8; [https://david.ncifcrf.gov/]) and Metascape (version 3.5; [https://metascape.org/]) to annotate DEPs and identify enriched pathways. Functional clustering highlighted mitochondrial metabolism, cytoskeletal organization, calcium signaling, and fibrosis-related processes. Comparative analysis of fibroblast and myocyte fractions revealed distinct remodeling signatures and therapeutic targets modulated by Vericiguat. These findings are consistent with prior reports of proteomic shifts in cardiac injury models [10] and support the mechanistic relevance of sGC stimulation in pressure-overload heart failure.

### 2.8. Statistical Analysis

Statistical analyses were performed using GraphPad Prism 9 (GraphPad Software, San Diego, CA, USA) or equivalent platforms. Data are expressed as mean ± standard deviation (SD), and normality was assessed before hypothesis testing. For comparisons among more than two groups, one-way analysis of variance (ANOVA) followed by Tukey’s post hoc test was applied. Direct pairwise comparisons were conducted using unpaired two-tailed Student’s *t*-tests. A *p*-value < 0.05 was considered statistically significant.

## 3. Results

### 3.1. Ventricular Wall Stress Assessment

**Figure 1:**
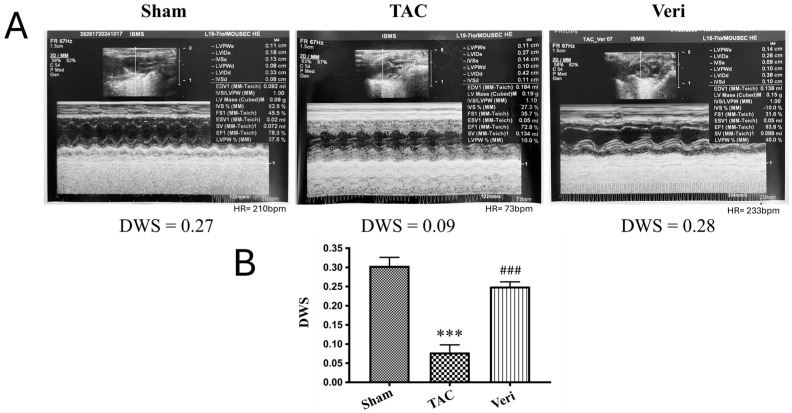
Vericiguat restores ventricular wall stress in pressure-overload heart failure. Representative M-mode echocardiographic images (**A**) and quantitative analysis of dynamic wall stress (DWS; (**B**)) are shown for Sham, transverse aortic constriction (TAC), and TAC + Vericiguat (Veri) groups. TAC significantly reduced DWS compared with Sham (*** *p* < 0.001), indicating impaired ventricular biomechanics. Vericiguat treatment restored DWS toward baseline (### *p* < 0.001 vs. TAC), reflecting improved contractility and normalization of wall stress regulation. Data are presented as mean ± SD; *n* = 6 per group.

Representative M-mode echocardiograms (Figure 1A) demonstrated distinct wall motion profiles across Sham-, TAC-, and Vericiguat-treated groups. TAC surgery markedly reduced DWS, indicating impaired biomechanics and elevated wall tension (*** *p* < 0.001 vs. Sham; Figure 1B). Vericiguat treatment restored DWS toward baseline (### *p* < 0.001 vs. TAC; Figure 1B), reflecting improved contractile performance and normalization of stress regulation. These results suggest that pressure overload disrupts ventricular mechanics, whereas soluble guanylate cyclase stimulation reverses this dysfunction and mitigates stress-induced remodeling.

### 3.2. Left Ventricular Remodeling and Function

TAC induced structural remodeling, as evidenced by increased diastolic mass, posterior wall thickness, and septal thickness compared with Sham (* *p* < 0.05 or *p* < 0.01; Table 1). Vericiguat attenuated these changes, reducing hypertrophic indices relative to untreated TAC (# *p* < 0.05 or ## *p* < 0.001). Functionally, TAC elevated end-systolic volume and reduced stroke volume, indicating systolic impairment (*p* < 0.05 or ** *p* < 0.01 vs. Sham). Vericiguat improved both parameters (# *p* < 0.05 or ## *p* < 0.001 vs. TAC) and partially normalized end-diastolic volume and internal diameters, supporting its role in preserving chamber geometry under hemodynamic stress.

### 3.3. Myocardial Hypertrophy and Fibrosis Assessment

**Figure 2:**
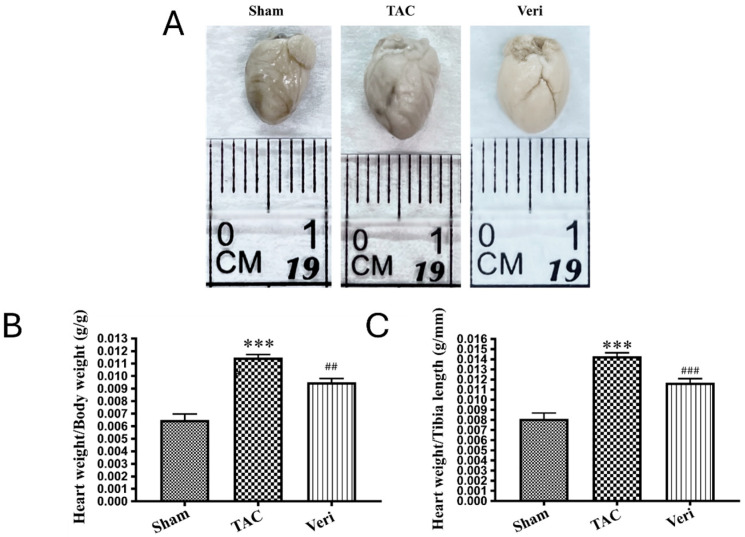
Vericiguat attenuates cardiac hypertrophy induced by pressure overload. Representative images of excised hearts (**A**) and quantification of heart weight normalized to body weight (**B**) and tibia length (**C**) are shown for Sham, transverse aortic constriction (TAC), and TAC + Vericiguat (Veri) groups. TAC induced significant cardiac enlargement compared with Sham (*** *p* < 0.001), whereas Vericiguat markedly reduced heart size and weight (## *p* < 0.01, ### *p* < 0.001 vs. TAC). Normalization to tibia length (**C**) was included to avoid potential bias introduced by post-surgical changes in body weight and to strengthen the robustness of hypertrophy assessment. Data are presented as mean ± SD; *n* = 6 per group.

**Figure 3:**
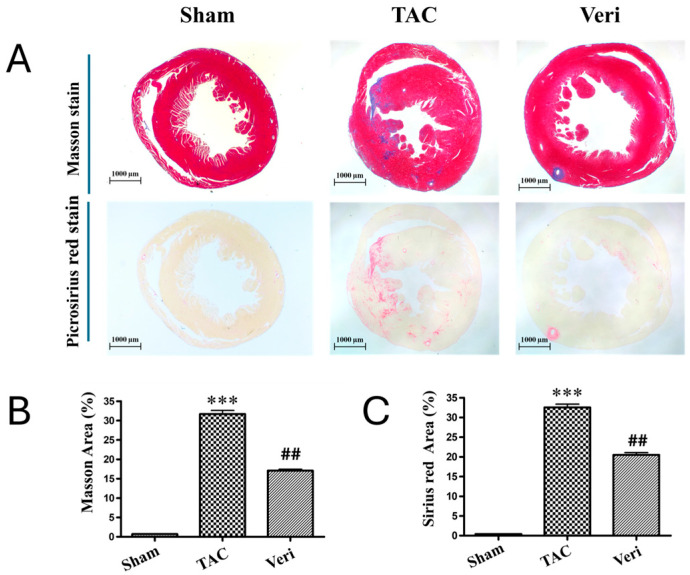
Vericiguat reduces myocardial fibrosis in pressure-overload heart failure. (**A**) Representative histological sections of left ventricular tissue stained with Masson’s trichrome (top row) and Picrosirius red (bottom row) from Sham, transverse aortic constriction (TAC), and TAC + Vericiguat (Veri) groups. Collagen deposition appears as blue in Masson’s trichrome and as red birefringence under polarized light in Picrosirius red. (**B**) Quantitative analysis of fibrotic area (%) from Masson’s trichrome and (**C**) Picrosirius red staining. TAC significantly increased myocardial fibrosis compared with Sham (*** *p* < 0.001), whereas Vericiguat markedly reduced fibrotic burden (## *p* < 0.01 vs. TAC). Data are presented as mean ± SD; *n* = 6 per group.

Gross morphological evaluation and heart weight quantification (Figure 2) revealed marked cardiac hypertrophy in mice subjected to TAC, which was significantly attenuated by Vericiguat treatment. Compared to Sham controls, TAC hearts exhibited increased size and weight (*** *p* < 0.001), while hearts from Vericiguat-treated animals showed intermediate values (## *p* < 0.01 vs. TAC), consistent with partial reversal of hypertrophic remodeling. Histological staining with Masson’s trichrome and Picrosirius red further demonstrated extensive collagen deposition in TAC hearts (Figure 3A), with significantly elevated fibrotic area relative to Sham (*** *p* < 0.001; Figure 3B,C). Vericiguat markedly reduced fibrosis in both assays (## *p* < 0.01 vs. TAC), indicating its efficacy in limiting extracellular matrix expansion. Collectively, these results support the dual antifibrotic and antihypertrophic effects of Vericiguat in pressure-overload-induced heart failure.

### 3.4. Fibroblast Proteome Reprogramming

**Figure 4:**
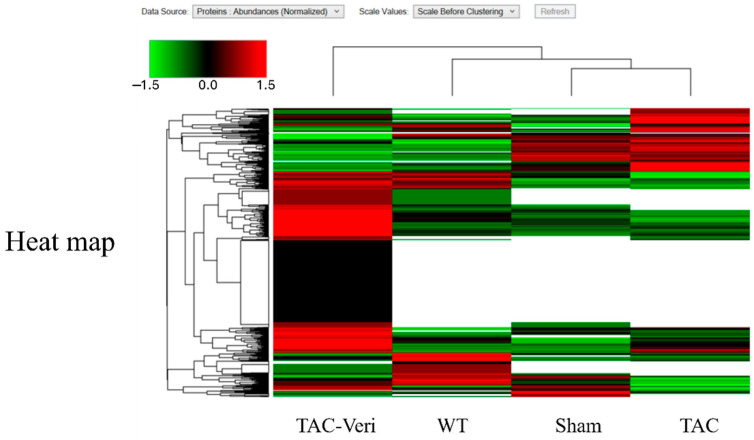
Vericiguat reprograms the fibroblast proteome in pressure-overload heart failure. Hierarchical heatmap of differentially expressed proteins (DEPs) in fibroblast-enriched cardiac tissue across four experimental groups: wild-type (WT), sham-operated (Sham), transverse aortic constriction (TAC), and TAC + Vericiguat (TAC-Ver). Protein abundance is color-coded (green: low; black: intermediate; red: high) and clustered by expression similarity. TAC markedly altered fibroblast proteomic profiles, with upregulation of pro-fibrotic and inflammatory markers and suppression of mitochondrial and reparative proteins. Vericiguat reversed these changes, restoring expression of bioenergetic, structural, and reparative components. These findings indicate that Vericiguat modulates fibroblast activity and extracellular matrix remodeling through targeted proteomic reprogramming.

Hierarchical clustering of DEPs in fibroblast-enriched cardiac tissue revealed distinct proteomic profiles across WT-, Sham-, TAC-, and Vericiguat-treated groups (Figure 4). TAC upregulated pro-fibrotic and inflammatory markers while downregulating mitochondrial and reparative proteins. Vericiguat reversed these alterations, restoring expression of bioenergetic and structural components. DEPs were functionally categorized into protein synthesis, folding, degradation, mitochondrial metabolism, and regulatory processes (Table 2). Notably, Vericiguat enhanced mitochondrial enzyme expression and translational machinery, suggesting improved protein homeostasis and extracellular matrix regulation. These findings highlight Vericiguat’s capacity to reprogram fibroblast activity and counteract maladaptive remodeling.

### 3.5. Myocyte Proteomic Remodeling

**Figure 5:**
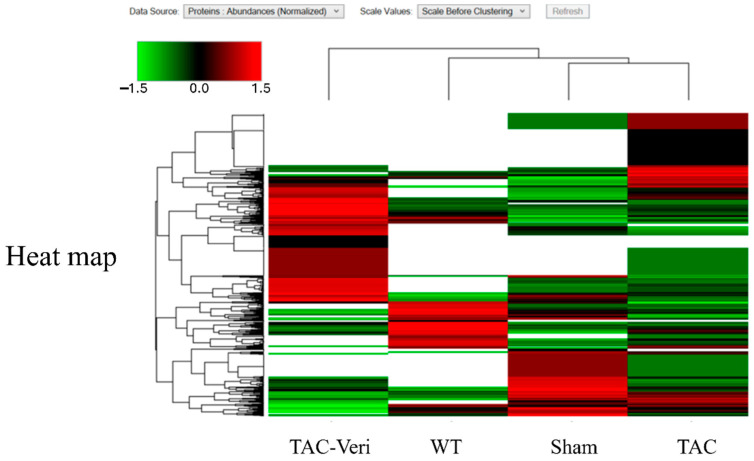
Vericiguat modulates myocyte proteomic signatures in pressure-overload heart failure. Hierarchical heatmap of differentially expressed proteins (DEPs) in myocyte-enriched cardiac tissue across four groups: wild-type (WT), sham-operated (Sham), transverse aortic constriction (TAC), and TAC + Vericiguat (TAC-Ver). Protein abundance is color-coded (green: low; black: intermediate; red: high) and clustered by expression similarity. TAC-induced pressure overload disrupted myocyte proteomic profiles, with downregulation of mitochondrial enzymes, contractile proteins, and calcium-handling components. Vericiguat reversed these alterations, restoring expression of bioenergetic and structural proteins. These findings suggest that Vericiguat preserves myocyte integrity and function through targeted proteomic reprogramming under pathological stress.

Proteomic analysis of myocyte-enriched tissue revealed that TAC disrupted the expression of mitochondrial enzymes, contractile proteins, and calcium-handling components (Figure 5). Vericiguat restored these profiles toward baseline, indicating recovery of cellular integrity. Functional classification of DEPs encompassed metabolic pathways, protein synthesis and modification, cytoskeletal and neuronal elements, mitochondrial regulators, transcriptional control, and vesicular transport (Table 3). Upregulation of mitochondrial and translational components, along with normalization of cytoskeletal and calcium-regulatory proteins, underscores Vericiguat’s role in preserving contractile architecture and intracellular signaling. These data emphasize its multi-pathway engagement in maintaining myocyte function under pathological stress.

## 4. Discussion

This study demonstrates that Vericiguat exerts multifaceted therapeutic effects in a pressure-overload model of heart failure, including attenuation of myocardial fibrosis, restoration of biomechanical integrity, and reversal of proteomic dysregulation. Histological analysis showed that Vericiguat markedly reduced collagen deposition in the left ventricle, as evidenced by Masson’s trichrome and Picrosirius red staining. These findings align with prior reports of Vericiguat suppressing extracellular matrix expansion and fibrotic remodeling in cardiac injury models such as doxorubicin-induced cardiomyopathy and atrial fibrillation [8,10]. The reduction in fibrotic burden is consistent with its modulation of TGF-β1/Smad2/3 signaling, a pathway central to collagen synthesis and fibroblast activation [8]. Functionally, Vericiguat improved wall stress indices and preserved ventricular geometry, suggesting enhanced compliance and contractile performance. These biomechanical improvements mirror clinical observations from the VICTORIA and VICTOR trials, where Vericiguat reduced cardiovascular events and supported hemodynamic stability in patients with heart failure and reduced ejection fraction [3,4]. Structural recovery in this preclinical model reinforces the hypothesis that Vericiguat’s benefits extend beyond symptomatic relief to direct modulation of cardiac architecture. Proteomic profiling further clarified its mechanistic impact, revealing reversal of TAC-induced suppression of mitochondrial, metabolic, and cytoskeletal proteins in fibroblast- and myocyte-enriched fractions. Notably, Vericiguat restored bioenergetic regulators and structural components, suggesting improved cellular resilience and energy handling. These findings are supported by prior studies demonstrating enhanced mitochondrial integrity and reduced oxidative stress via AMPK/Nrf2/NLRP3 signaling [7,9]. Collectively, histological, functional, and proteomic data underscore Vericiguat’s ability to mitigate pressure-overload remodeling through antifibrotic, biomechanical, and metabolic reprogramming mechanisms. This comprehensive profile supports its continued development as a structurally restorative agent in heart failure management.

Analysis of echocardiographic parameters revealed distinct remodeling patterns across groups. The interventricular septum (IVS) % change provided insight into adaptive versus maladaptive remodeling. Sham animals exhibited physiological septal thickening (~50%), reflecting normal contractile dynamics. In contrast, TAC abolished this adaptive response (0%), consistent with pathological stiffening and loss of flexibility under pressure overload. Vericiguat partially restored septal dynamics, though the negative value observed suggests regression of maladaptive hypertrophy rather than conventional thickening, highlighting its role in remodeling reversal [1]. Posterior wall (LVPW) % change served as an indicator of contractile reserve. Sham mice demonstrated robust thickening (~37%), whereas TAC abolished this response, reflecting impaired reserve under sustained afterload stress. Vericiguat restored posterior wall dynamics (~40%), consistent with its antihypertrophic and antifibrotic effects, supporting improved myocardial adaptability under pressure overload [10]. Heart rate (HR) differences observed in M-mode imaging further contextualized these findings. TAC animals displayed compensatory tachycardia, a common response to increased afterload and reduced stroke volume. Vericiguat partially normalized HR, suggesting improved hemodynamic adaptation and reduced reliance on maladaptive mechanisms [9]. Interestingly, ejection fraction (EF) remained similar across groups, indicating preserved global systolic function at this early stage of remodeling. This underscores that Vericiguat’s primary benefit lies not in altering EF, but in mitigating structural remodeling, reducing wall stress, and restoring biomechanical indices. Such effects are particularly relevant in early-stage heart failure, where structural changes precede overt declines in systolic function [2]. Together, these observations highlight Vericiguat’s capacity to normalize wall stress and reverse maladaptive remodeling without altering global EF. By improving septal flexibility, restoring posterior wall contractile reserve, and stabilizing hemodynamic responses, Vericiguat demonstrates a multifaceted therapeutic profile in pressure-overload heart failure.

The present findings complement and extend prior evidence supporting Vericiguat’s role in attenuating pathological cardiac remodeling through antifibrotic and mitochondrial protective mechanisms. In coronary microembolization models, Vericiguat reduced pyroptosis and inflammatory signaling by modulating the AMPK/Nrf2/NLRP3 axis, thereby limiting myocardial injury and preserving cellular resilience [7]. Similarly, studies in atrial tissue showed that Vericiguat alleviates fibrosis by suppressing TGF-β1/Smad2/3 signaling, a pathway also implicated in extracellular matrix expansion under pressure-overload conditions [8]. These mechanistic parallels reinforce the drug’s ability to influence key remodeling cascades across diverse cardiac contexts. Importantly, our work provides direct histological and proteomic evidence of Vericiguat’s efficacy in a TAC model, which recapitulates hypertensive and valvular stress. Large clinical trials such as VICTORIA and VICTOR confirmed Vericiguat’s benefit in reducing cardiovascular death and hospitalization in patients with heart failure and reduced ejection fraction [3,4], but did not assess its impact on myocardial architecture or cell-type-specific remodeling. Our data address this gap by showing that Vericiguat improves wall stress regulation, preserves ventricular geometry, and reverses TAC-induced proteomic dysregulation in both fibroblasts and myocytes. Comparisons with other injury models, including doxorubicin-induced cardiomyopathy and ischemia–reperfusion injury, suggest that Vericiguat consistently supports mitochondrial integrity, reduces oxidative stress, and stabilizes cytoskeletal organization [9,10]. Nevertheless, the proteomic signatures identified here highlight context-dependent effects. Restoration of mitochondrial proteins was a common feature across models, whereas modulation of calcium-handling and cytoskeletal proteins was more pronounced in the TAC setting, likely reflecting the biomechanical demands of pressure overload. Taken together, these comparisons underscore Vericiguat’s versatility in targeting both shared and condition-specific pathways of cardiac remodeling. By integrating histological, functional, and proteomic endpoints, our study advances mechanistic understanding of Vericiguat and supports its broader application in structurally compromised heart failure phenotypes, while also emphasizing the need for continued evaluation of safety and subgroup responses in clinical practice.

The therapeutic effects of Vericiguat observed in this study appear to involve modulation of multiple signaling pathways implicated in cardiac fibrosis, inflammation, and bioenergetic dysfunction. Notably, Vericiguat attenuated pressure-overload remodeling through suppression of the TGF-β1/Smad2/3 axis, a central driver of fibroblast activation and collagen synthesis. This pathway has been linked to atrial fibrosis and arrhythmogenic remodeling, and its downregulation by Vericiguat reduces extracellular matrix expansion and improves tissue architecture [8]. In parallel, Vericiguat inhibited components of the AMPK/Nrf2/NLRP3 cascade, which regulates oxidative stress and pyroptotic cell death. In coronary microembolization models, activation of AMPK and Nrf2 suppressed NLRP3 inflammasome activity, thereby reducing inflammatory injury and preserving myocardial integrity [7]. Our findings extend this mechanism to pressure-overload conditions, suggesting that Vericiguat’s anti-inflammatory effects may be broadly relevant across ischemic and hypertensive heart failure phenotypes. Emerging evidence also points to a possible interaction with CaMKII-related signaling, which governs intracellular calcium dynamics and contributes to maladaptive remodeling and arrhythmogenesis. Although direct modulation of CaMKII was not confirmed here, proteomic shifts in calcium-handling proteins and cytoskeletal regulators raise the hypothesis that Vericiguat may stabilize excitation–contraction coupling and mitigate calcium-driven stress responses [1]. Proteomic profiling revealed distinct patterns of DEPs in fibroblast and myocyte populations, highlighting cell-type-specific responses. In fibroblasts, Vericiguat reversed TAC-induced upregulation of pro-fibrotic and cytoskeletal proteins while restoring mitochondrial and structural components. In myocytes, treatment enhanced metabolic enzymes and mitochondrial regulators, consistent with improved bioenergetic support and contractile function. These findings align with prior reports showing Vericiguat’s ability to preserve mitochondrial quality and reduce oxidative damage in models of doxorubicin-induced cardiomyopathy and mitral regurgitation [7,10]. Together, these mechanistic insights underscore Vericiguat’s multifactorial role in cardiac remodeling. By targeting fibrosis, inflammation, calcium homeostasis, and metabolic resilience, Vericiguat supports structural recovery and functional improvement in pressure-overload heart failure. The cell-type-specific proteomic shifts further suggest that its therapeutic effects are coordinated across both stromal and contractile compartments, reinforcing its potential as a comprehensive modulator of myocardial remodeling.

The findings of this study reinforce Vericiguat’s emerging role as a dual-action therapeutic agent in HFrEF, offering both symptomatic relief and structural remodeling benefits. Large-scale clinical trials such as VICTORIA and VICTOR have demonstrated its ability to reduce cardiovascular mortality and heart failure-related hospitalizations in high-risk populations [3,4], Our data provide complementary mechanistic evidence, showing that Vericiguat mitigates myocardial fibrosis and restores ventricular architecture—addressing structural deterioration that often persists despite optimal neurohormonal blockade. The pressure-overload model employed here closely reflects hypertensive and valvular heart disease, conditions characterized by elevated wall stress, collagen accumulation, and progressive ventricular dysfunction. Because these pathologies often precede overt heart failure, our findings suggest that earlier intervention with Vericiguat may help delay disease progression. By demonstrating reversal of fibrotic remodeling and improvement in biomechanical indices, this study supports the concept that Vericiguat’s therapeutic window could extend beyond post-decompensation settings to patients with subclinical or early-stage structural compromise. This is particularly relevant given the limited efficacy of conventional agents in reversing fibrosis and chamber dilation [9,12]. Safety and tolerability remain central considerations in the adoption of novel therapies. Subgroup analyses from the VICTORIA trial demonstrated consistent benefit across patients with diabetes, renal impairment, and other comorbidities, without significant adverse hemodynamic effects [5,13]. Real-world evidence further supports these findings, showing that Vericiguat can be safely initiated even in patients with fluctuating renal function, thereby broadening its clinical applicability [5]. Beyond safety, recent investigations highlight Vericiguat’s mechanistic and functional advantages. Experimental studies indicate that soluble guanylate cyclase stimulation improves mitochondrial activity, reduces oxidative stress, and attenuates maladaptive remodeling in models of heart failure with preserved or reduced ejection fraction [9,12]. Clinical data also suggest that Vericiguat may reduce recurrent hospitalizations and improve short-term cardiovascular function, even in patients with advanced disease or concomitant renal dysfunction [5,14]. Additional reports describe its role in mitigating arrhythmia recurrence and chemotherapy-induced cardiotoxicity, underscoring a broader cardioprotective profile that extends beyond hemodynamic stabilization [8,15]. Taken together, evidence from randomized trials, real-world studies, and mechanistic investigations converges to support Vericiguat as a well-tolerated therapy with consistent efficacy across diverse patient populations. These insights enrich the dialog on its therapeutic potential, offering clinicians and researchers a more comprehensive understanding of its role in heart failure management. Our proteomic findings further align with these observations, highlighting restoration of mitochondrial and energy-related proteins in both fibroblast and myocyte populations. Such molecular reprogramming suggests that Vericiguat may be particularly beneficial in metabolically vulnerable subgroups, where impaired bioenergetic capacity contributes to poor outcomes. Collectively, these insights support a precision medicine approach in which proteomic or biomarker-guided stratification could optimize therapeutic outcomes. By targeting both functional symptoms and structural pathology, Vericiguat expands its clinical relevance in HFrEF. Its integration into earlier treatment phases, combined with targeted use in patients with high fibrotic burden or metabolic dysregulation, may enhance long-term outcomes and slow progression to advanced heart failure.

While this study provides compelling evidence for Vericiguat’s structural and molecular benefits in pressure-overload heart failure, several limitations should be acknowledged. First, the treatment duration was limited to 14 days post-TAC surgery, which may not fully capture the long-term trajectory of myocardial remodeling or sustained therapeutic effects. Previous studies have shown that chronic administration of Vericiguat can influence survival and progressive remodeling in models of volume overload and cardiotoxicity [10,16], suggesting that extended observation periods may reveal additional benefits or compensatory changes. Second, although the murine TAC model reproduces key features of hypertensive and valvular heart disease, including elevated wall stress and fibrotic remodeling, it does not fully reflect the complexity of human heart failure. Factors such as comorbid metabolic dysfunction, neurohormonal activation, and sex-dependent susceptibility are variably represented in animal models [17], and translational caution is warranted when extrapolating these findings to clinical populations. Third, while proteomic profiling revealed modulation of several signaling pathways, including TGF-β1/Smad2/3 and AMPK/Nrf2/NLRP3, direct causal validation was not performed. The potential involvement of CaMKII-related signaling—implicated in calcium dysregulation and arrhythmogenic remodeling—remains speculative. Although prior reports suggest Vericiguat may stabilize intracellular calcium dynamics [1], targeted studies using pathway-specific inhibitors or genetic knockouts are needed to confirm these mechanistic links. Future investigations should incorporate longer treatment windows, multi-organ assessments, and mechanistic interventions to delineate Vericiguat’s full therapeutic profile. Integration of genetic models and pharmacological modulators will be essential to validate pathway-specific effects and refine patient stratification strategies for clinical application.

Building on the mechanistic and histological insights presented in this study, several avenues for future investigation are warranted to further define and translate the therapeutic potential of Vericiguat in heart failure. First, longitudinal studies are needed to assess the durability of its anti-remodeling effects. While short-term improvements in fibrosis and proteomic profiles were evident, extended treatment protocols may reveal sustained benefits or compensatory adaptations in myocardial structure and function. Prior work in volume-overload and cardiotoxicity models has shown that prolonged sGC stimulation improves survival and attenuates progressive remodeling [10,16], supporting the rationale for chronic-phase evaluation. Second, validation of DEPs and signaling targets identified through proteomic analysis should be pursued using orthogonal methods such as Western blotting and immunohistochemistry. This will confirm the involvement of key pathways—including TGF-β1/Smad2/3, AMPK/Nrf2/NLRP3, and potentially CaMKII—and clarify their spatial and cell-type-specific activation patterns. Such mechanistic validation is essential for establishing causal links and identifying therapeutic biomarkers. Third, exploration of Vericiguat’s efficacy in comorbid models is critical for clinical relevance. Heart failure often coexists with metabolic and renal dysfunction, which may alter drug responsiveness. Recent studies have shown that Vericiguat maintains cardiovascular benefits in patients with impaired renal function and diabetes [5,6], but preclinical models incorporating these comorbidities could provide deeper insight into molecular adaptations and safety profiles. Finally, clinical translation would benefit from biomarker-guided stratification strategies to identify patients most likely to respond to sGC stimulation. Proteomic signatures, circulating fibrosis markers, or mitochondrial stress indicators may serve as predictive tools for tailoring therapy. This approach aligns with emerging precision medicine frameworks and could enhance the therapeutic index of Vericiguat in diverse heart failure populations [1,18]. In summary, future studies should integrate longitudinal design, mechanistic validation, comorbidity modeling, and translational biomarker development to fully define Vericiguat’s role in heart failure management and optimize its clinical deployment.

In conclusion, this study demonstrates that Vericiguat exerts robust anti-remodeling effects in a pressure-overload model of heart failure, characterized by reduced myocardial fibrosis, improved ventricular geometry, and reversal of proteomic dysregulation in fibroblast and myocyte populations. By modulating key signaling pathways—including TGF-β1/Smad2/3 and AMPK/Nrf2/NLRP3—Vericiguat mitigates fibrotic and inflammatory responses while supporting mitochondrial and structural integrity. These findings extend prior evidence from ischemic and cardiotoxic models [7,8,10] and address a mechanistic gap left by clinical trials such as VICTORIA and VICTOR, which primarily focused on symptomatic endpoints [3,4]. Importantly, the cell-type-specific proteomic shifts observed here highlight Vericiguat’s capacity to coordinate stromal and contractile recovery, suggesting potential utility in patient subgroups with high fibrotic burden or metabolic vulnerability. These insights support repositioning Vericiguat as a dual-action agent, capable of both symptom relief and structural remodeling, and underscore the need for biomarker-guided strategies to optimize clinical deployment. Future studies should incorporate longer treatment durations, mechanistic validation with pathway-specific inhibitors, and comorbid models to further define Vericiguat’s therapeutic scope. Collectively, these findings reinforce the translational relevance of sGC stimulation in heart failure and provide a foundation for precision-guided intervention in structurally compromised cardiac phenotypes.

## Figures and Tables

**Table 1:** Echocardiographic parameters following vericiguat treatment in TAC-induced cardiac hypertrophy.

	Sham	TAC	Veri
LVEDV (mL)	0.131 ± 0.023	0.241 ± 0.086 *	0.124 ± 0.037 ^#^
LVESV (mL)	0.034 ± 0.012	0.086 ± 0.037 *	0.038 ± 0.019 ^#^
SV (mL)	0.097 ± 0.019	0.155 ± 0.050 *	0.086 ± 0.019 ^#^
LVd Mass	0.104 ± 0.010	0.204 ± 0.031 ***	0.150 ± 0.018 ^##^
LVIDs	0.226 ± 0.031	0.318 ± 0.045 **	0.234 ± 0.041 ^##^
LVIDd	0.372 ± 0.024	0.456 ± 0.055 *	0.362 ± 0.038 ^#^
LVPWd	0.080 ± 0.000	0.096 ± 0.010 **	0.100 ± 0.000 ^#^
IVSs	0.116 ± 0.010	0.122 ± 0.012	0.138 ± 0.028
IVSd	0.080 ± 0.000	0.108 ± 0.007 ***	0.114 ± 0.012

Summary of left ventricular structural and functional indices measured by echocardiography in three experimental groups: Sham, transverse aortic constriction (TAC), and TAC + Vericiguat (Veri). Parameters include end-diastolic volume (LVEDV), end-systolic volume (LVESV), stroke volume (SV), diastolic mass (LVd Mass), internal diameters (LVIDs, LVIDd), posterior wall thickness (LVPWd), interventricular septal thickness at end-diastole (IVSd), and interventricular septal thickness at end-systole (IVSs). Data are presented as mean ± SD; n = 6 per group. * *p* < 0.05 vs. Sham; ** *p* < 0.01 vs. Sham; *** *p* < 0.001 vs. Sham; # *p* < 0.05 vs. TAC; ## *p* < 0.01 vs. TAC.

**Table 2:** Functional classification of differentially expressed proteins in TAC vs. Vericiguat comparison (fibroblast-enriched fraction).

**Accession ID**	Protein Name	WTC Abundance Ratio	STC Abundance Ratio	TTVeri Abundance Ratio	*p*-Value
**Metabolism and Energy-Related**					
P53395	Lipoamide acyltransferase component of branched-chain alpha-keto acid dehydrogenase complex, mitochondrial	0.01	0.01	100	1 × 10^−17^
Q9CXJ1	Nondiscriminating glutamyl-tRNA synthetase EARS2, mitochondrial	0.01	0.01	100	1 × 10^−17^
P32020	Sterol carrier protein 2	0.01	0.01	100	1 × 10^−17^
A2APY7	rginine-hydroxylase NDUFAF5, mitochondrial	0.01	0.01	100	1 × 10^−17^
P17182	Alpha-enolase	0.01	0.01	100	1 × 10^−17^
P06745	Glucose-6-phosphate isomerase	0.01	0.01	100	1 × 10^−17^
Q8R3F5	Malonyl-CoA-acyl carrier protein transacylase, mitochondrial	0.01	0.01	100	1 × 10^−17^
Q9CQ43	Deoxyuridine 5′-triphosphate nucleotidohydrolase	0.01	0.01	100	1 × 10^−17^
Q8K009	Mitochondrial 10-formyltetrahydrofolate dehydrogenase	0.01	0.01	100	1 × 10^−17^
O88986	2-amino-3-ketobutyrate coenzyme A ligase, mitochondrial	0.01	0.01	100	1 × 10^−17^
Q3UUI3	Acyl-coenzyme A thioesterase THEM4	0.01	0.01	100	1 × 10^−17^
Q9D009	Octanoyl-[acyl-carrier-protein]:protein N-octanoyltransferase LIPT2, mitochondrial	0.01	0.01	100	1 × 10^−17^
P17563	Methanethiol oxidase	0.01	0.01	100	1 × 10^−17^
Q9EQ06	Estradiol 17-beta-dehydrogenase 11	0.01	0.01	100	1 × 10^−17^
Q9CQX8	Alpha-ketoglutarate dehydrogenase component 4	0.01	0.01	100	1 × 10^−17^
Q9QZF2	Glypican-1	0.01	0.01	100	1 × 10^−17^
A0A1B0GR11	Transaldolase	0.01	0.01	100	1 × 10^−17^
P98192	Dihydroxyacetone phosphate acyltransferase	0.01	0.01	100	1 × 10^−17^
P11152	Lipoprotein lipase	0.01	0.01	100	1 × 10^−17^
**Protein Synthesis and Modification**					
P68040	Small ribosomal subunit protein RACK1	0.01	0.01	100	1 × 10^−17^
Q9DAT5	Mitochondrial tRNA-specific 2-thiouridylase 1	0.01	0.01	100	1 × 10^−17^
Q9CQ40	Large ribosomal subunit protein mL49	0.01	0.01	100	1 × 10^−17^
Q9DCI9	Large ribosomal subunit protein bL32m	0.01	0.01	100	1 × 10^−17^
Q9D2R8	Small ribosomal subunit protein mS33	0.01	0.01	100	1 × 10^−17^
P83882	Large ribosomal subunit protein eL42	0.01	0.01	100	1 × 10^−17^
P62264	Small ribosomal subunit protein uS11	0.01	0.01	100	1 × 10^−17^
P15532	Nucleoside diphosphate kinase A	0.01	0.01	100	1 × 10^−17^
**Structural and Cytoskeletal**					
Q8BFZ3	Beta-actin-like protein 2	0.01	0.01	100	1 × 10^−17^
D3YUI7	Myosin regulatory light chain 2, ventricular/cardiac muscle isoform (Fragment)	0.01	0.01	100	1 × 10^−17^
F8VQJ3	Laminin, gamma 1	0.01	0.01	100	1 × 10^−17^
P82348	Gamma-sarcoglycan	100	100	0.01	1 × 10^−17^
Q02788	Collagen alpha-2(VI) chain	0.01	0.01	100	1 × 10^−17^
P47754	F-actin-capping protein subunit alpha-2	100	100	0.01	1 × 10^−17^
**Mitochondrial Function**					
A0A0R4J0T0	HscB iron-sulfur cluster co-chaperone	0.01	0.01	100	1 × 10^−17^
O35943	Frataxin, mitochondrial	0.01	0.01	100	1 × 10^−17^
Q99P30	Peroxisomal coenzyme A diphosphatase NUDT7	0.01	0.01	100	1 × 10^−17^
Q9DCB8	Iron-sulfur cluster assembly 2 homolog, mitochondrial	0.01	0.01	100	1 × 10^−17^
P56501	Putative mitochondrial transporter UCP3	0.01	0.01	100	1 × 10^−17^
Q8BUY5	Complex I assembly factor TIMMDC1, mitochondrial	0.01	0.01	100	1 × 10^−17^
**Signal Transduction and Regulation**					
Q3TL44	NLR family member X1	0.01	0.01	100	1 × 10^−17^
A0A1B0GRP7	Pyridoxal phosphate binding protein	0.01	0.01	100	1 × 10^−17^
Q76I26	Methyltransferase hypoxia inducible domain containing 1	0.01	0.01	100	1 × 10^−17^
Q8BXN7	Protein phosphatase Mn(2+)-dependent 1K	0.01	0.01	100	1 × 10^−17^
Q99LX0	Parkinson disease protein 7 homolog	0.01	0.01	100	1 × 10^−17^
P47199	Quinone oxidoreductase	0.01	0.01	100	1 × 10^−17^
Q8VDK1	Deaminated glutathione amidase	0.01	0.01	100	1 × 10^−17^
Q6YI28	EP1	0.01	0.01	100	1 × 10^−17^
**Transport and Translocation**					
Q8BJS4	SUN domain-containing protein 2	0.01	0.01	100	1 × 10^−17^
Q9D273	Corrinoid adenosyltransferase MMAB	0.01	0.01	100	1 × 10^−17^
Q5SWT3	Solute carrier family 25 member 35	0.01	0.01	100	1 × 10^−17^
Q3URS9	Mitochondrial potassium channel	0.01	0.01	100	1 × 10^−17^

Differentially expressed proteins (DEPs) were identified in mice subjected to transverse aortic constriction without (TAC) or with Vericiguat treatment (Veri), selected based on log_2_ fold change < 0.58 and *p* < 0.001. Proteins are grouped by biological function, including protein synthesis (ribosomal subunits, translation initiation/elongation factors, aminoacyl-tRNA synthetases), protein folding (molecular chaperones), degradation (proteasome subunits, ubiquitin-related enzymes), mitochondrial metabolism, and other regulatory processes. Vericiguat treatment consistently upregulated mitochondrial enzymes and translational machinery, indicating restoration of bioenergetic and biosynthetic capacity. These proteomic shifts support enhanced protein homeostasis and structural integrity, underscoring Vericiguat’s multi-pathway role in reversing pressure-overload–induced myocardial remodeling.

**Table 3:** Functional classification of differentially expressed proteins in TAC vs. Vericiguat comparison (myocyte-enriched fraction).

Accession ID	Protein Name	WTC Abundance Ratio	STC Abundance Ratio	TTVeri Abundance Ratio	***p*-Value**
**Metabolism and Energy-Related**					
A0A338P776	ATP synthase subunit O	0.01	0.01	100	1 × 10^−17^
A0A2R8VJW0	Aconitase 2, mitochondrial	0.01	0.01	100	1 × 10^−17^
O08528	Hexokinase-2	0.01	0.01	100	1 × 10^−17^
P11152	Lipoprotein lipase	0.01	0.01	100	1 × 10^−17^
Q64516	Glycerol kinase	0.01	0.01	100	1 × 10^−17^
Q9QX60	Deoxyguanosine kinase, mitochondrial	0.01	0.01	100	1 × 10^−17^
Q8R3F5	Malonyl-CoA-acyl carrier protein transacylase	0.01	0.01	100	1 × 10^−17^
Q8BHF7	CDP-diacylglycerol--glycerol-3-phosphate 3-phosphatidyltransferase, mitochondrial	0.01	0.01	100	1 × 10^−17^
A0A0R4J135	Methanethiol oxidase	0.01	0.01	100	1 × 10^−17^
Q9Z0K8	Pantetheinase	0.01	0.01	100	1 × 10^−17^
Q9Z1J3	Cysteine desulfurase	0.01	0.01	100	1 × 10^−17^
P32020	Sterol carrier protein 2	0.01	0.01	100	1 × 10^−17^
A2AL50	Alkylglycerone-phosphate synthase	0.01	0.01	100	1 × 10^−17^
Q8BP40	Lysophosphatidic acid phosphatase type 6	0.01	0.01	100	1 × 10^−17^
Q9CQ43	Deoxyuridine 5′-triphosphate nucleotidohydrolase	0.01	0.01	100	1 × 10^−17^
Q99L04	Dehydrogenase/reductase SDR family member 1	0.01	0.01	100	1 × 10^−17^
D3YU39	Cholinephosphotransferase 1	0.01	0.01	100	1 × 10^−17^
**Protein Synthesis and Modification**					
Q8BU88	Large ribosomal subunit protein uL22m	0.01	0.01	100	1 × 10^−17^
Q9D773	Large ribosomal subunit protein uL2m	0.01	0.01	100	1 × 10^−17^
Q9CXW2	Small ribosomal subunit protein mS22	0.01	0.01	100	1 × 10^−17^
Q9CXJ1	Nondiscriminating glutamyl-tRNA synthetase EARS2	0.01	0.01	100	1 × 10^−17^
Q9DB15	Large ribosomal subunit protein bL12m	0.01	0.01	100	1 × 10^−17^
P62918	Large ribosomal subunit protein uL2	0.01	0.01	100	1 × 10^−17^
A0A286YCZ5	phenylalanine-tRNA ligase	0.01	0.01	100	1 × 10^−17^
Q924T2	Small ribosomal subunit protein uS2m	0.01	0.01	100	1 × 10^−17^
Q6ZWV7	Large ribosomal subunit protein uL29	0.01	0.01	100	1 × 10^−17^
Q9DAT5	Mitochondrial tRNA-specific 2-thiouridylase 1	0.01	0.01	100	1 × 10^−17^
P61255	Large ribosomal subunit protein uL24	0.01	0.01	100	1 × 10^−17^
P46978	Dolichyl-diphosphooligosaccharide--protein glycosyltransferase subunit STT3A	0.01	0.01	100	1 × 10^−17^
Q8BL63	GPI-anchor transamidase	0.01	0.01	100	1 × 10^−17^
Q9CPR4	Large ribosomal subunit protein uL22	0.01	0.01	100	1 × 10^−17^
P27659	Large ribosomal subunit protein uL3	0.01	0.01	100	1 × 10^−17^
P62852	Small ribosomal subunit protein eS25	0.01	0.01	100	1 × 10^−17^
O08832	Polypeptide N-acetylgalactosaminyltransferase 4	0.01	0.01	100	1 × 10^−17^
**Structural and Cytoskeletal**					
Q5SX40	Myosin-1	0.01	0.01	100	1 × 10^−17^
Q9Z2T6	Keratin, type II cuticular Hb5	0.01	0.01	100	1 × 10^−17^
Q8BFZ3	Beta-actin-like protein 2	0.01	0.01	100	1 × 10^−17^
A2AQP0	Myosin-7B	0.01	0.01	100	1 × 10^−17^
Q8K0Y2	Keratin, type I cuticular Ha3-I	0.01	0.01	100	1 × 10^−17^
P05977	Myosin light chain 1/3, skeletal muscle isoform	0.01	0.01	100	1 × 10^−17^
Q61554	Fibrillin-1	0.01	0.01	100	1 × 10^−17^
P26231	Catenin alpha-1	0.01	0.01	100	1 × 10^−17^
Q64291	Keratin, type I cytoskeletal 12	0.01	0.01	100	1 × 10^−17^
Q02788	Collagen alpha-2(VI) chain	0.01	0.01	100	1 × 10^−17^
Q9R1Q7	Proteolipid protein 2	0.01	0.01	100	1 × 10^−17^
**Mitochondrial Function**					
Q6PE15	Palmitoyl-protein thioesterase ABHD10	0.01	0.01	100	1 × 10^−17^
Q3U276	Succinate dehydrogenase assembly factor 1	0.01	0.01	100	1 × 10^−17^
O88696	ATP-dependent Clp protease proteolytic subunit	0.01	0.01	100	1 × 10^−17^
I3ITR1	Iron-sulfur cluster assembly 1 homolog	0.01	0.01	100	1 × 10^−17^
Q99JB2	Stomatin-like protein 2	0.01	0.01	100	1 × 10^−17^
Q8BGX2	Mitochondrial import inner membrane translocase subunit Tim29	0.01	0.01	100	1 × 10^−17^
Q59J78	NADH dehydrogenase [ubiquinone] 1 alpha subcomplex assembly factor 2	0.01	0.01	100	1 × 10^−17^
Q8BG51	Mitochondrial Rho GTPase 1	0.01	0.01	100	1 × 10^−17^
**Signal Transduction and Regulation**					
Q02819	Nucleobindin-1	0.01	0.01	100	1 × 10^−17^
P63101	14-3-3 protein zeta/delta	0.01	0.01	100	1 × 10^−17^
O08795	Glucosidase 2 subunit beta	0.01	0.01	100	1 × 10^−17^
A0A1B0GRP7	Pyridoxal phosphate binding protein	0.01	0.01	100	1 × 10^−17^
Q9QZF2	Glypican-1	0.01	0.01	100	1 × 10^−17^
P97443	Histone-lysine N-methyltransferase Smyd1	0.01	0.01	100	1 × 10^−17^
Q8BTE5	Protein CEBPZOS	0.01	0.01	100	1 × 10^−17^
Q99LX0	Parkinson disease protein 7 homolog	0.01	0.01	100	1 × 10^−17^
Q8R0J2	Mannose-P-dolichol utilization defect 1 protein	0.01	0.01	100	1 × 10^−17^
P17426	AP-2 complex subunit alpha-1	0.01	0.01	100	1 × 10^−17^
Q8BTJ4	Bis(5′-adenosyl)-triphosphatase enpp4	0.01	0.01	100	1 × 10^−17^
Q9Z2L6	Multiple inositol polyphosphate phosphatase 1	0.01	0.01	100	1 × 10^−17^
P15532	Nucleoside diphosphate kinase A	0.01	0.01	100	1 × 10^−17^
P01027	Complement C3	0.01	0.01	100	1 × 10^−17^
A0A0M3HEQ0	thioredoxin-disulfide reductase	0.01	0.01	100	1 × 10^−17^
P11352	Glutathione peroxidase 1	0.01	0.01	100	1 × 10^−17^
P21460	Cystatin-C	0.01	0.01	100	1 × 10^−17^
Q91X72	Hemopexin	0.01	0.01	100	1 × 10^−17^
Q9CRY7	Lysophospholipase D GDPD1	0.01	0.01	100	1 × 10^−17^
Q8C0L0	Thioredoxin-related transmembrane protein 4	0.01	0.01	100	1 × 10^−17^
Q8K1N1	Calcium-independent phospholipase A2-gamma	0.01	0.01	100	1 × 10^−17^
Q9CPS6	Adenosine 5′-monophosphoramidase HINT3	0.01	0.01	100	1 × 10^−17^
O88342	WD repeat-containing protein 1	0.01	0.01	100	1 × 10^−17^
A0A0R4J0Z1	Protein disulfide-isomerase A4	0.01	0.01	100	1 × 10^−17^
Q9CYW4	Haloacid dehalogenase-like hydrolase domain-containing protein 3	0.01	0.01	100	1 × 10^−17^
Q61171	Peroxiredoxin-2	0.01	0.01	100	1 × 10^−17^
**Transport and Translocation**					
P53810	Phosphatidylinositol transfer protein alpha isoform	0.01	0.01	100	1 × 10^−17^
Q99KF1	Transmembrane emp24 domain-containing protein 9	0.01	0.01	100	1 × 10^−17^
P58021	Transmembrane 9 superfamily member 2	0.01	0.01	100	1 × 10^−17^
Q9EQH3	Vacuolar protein sorting-associated protein 35	0.01	0.01	100	1 × 10^−17^
Q8R1V4	Transmembrane emp24 domain-containing protein 4	0.01	0.01	100	1 × 10^−17^
Q8BPE4	Transmembrane protein 177	0.01	0.01	100	1 × 10^−17^
Q9ET30	Transmembrane 9 superfamily member 3	0.01	0.01	100	1 × 10^−17^
Q99P72	Reticulon-4	0.01	0.01	100	1 × 10^−17^
Q9CXE7	Transmembrane emp24 domain-containing protein 5	0.01	0.01	100	1 × 10^−17^
O08579	Emerin	0.01	0.01	100	1 × 10^−17^
Q3U3G8	Serine hydrolase-like	0.01	0.01	100	1 × 10^−17^
P17047	Lysosome-associated membrane glycoprotein 2	0.01	0.01	100	1 × 10^−17^

Differentially expressed proteins (DEPs) were identified in mice subjected to transverse aortic constriction without (TAC) or with Vericiguat treatment (Vericiguat), selected based on log_2_ fold change < 0.58 and *p* < 0.001. Proteins are grouped by biological function, including metabolism and energy production, protein synthesis and modification, cytoskeletal and neuronal components, mitochondrial regulators, transcriptional control, and vesicular transport. Vericiguat treatment consistently upregulated mitochondrial enzymes and translational machinery, indicating restoration of bioenergetic and biosynthetic capacity. These proteomic shifts support improved protein homeostasis and structural integrity, underscoring Vericiguat’s multi-pathway role in reversing pressure-overload-induced myocardial remodeling.

## Data Availability

The original contributions presented in this study are included in the article/Supplementary Materials. Further inquiries can be directed to the corresponding author.

## Supplementary Materials

The following supporting information can be downloaded at: https://www.mdpi.com/article/10.3390/life15111763/s1. Figure S1: Pressure–Volume Loop Analysis in the Pressure-Overload Model.
