# Supplementary material for: Therapeutic Impact of Vericiguat on Ventricular Remodeling in a Pressure-Overload Heart Failure Model
Source: Life (Basel). 2025 Nov 18;15(11):1763. doi: 10.3390/life15111763 (PMC12653654; doi:10.3390/life15111763)
Supplement: Supplementary file 1 [file life-15-01763-s001.zip › life-3992658-supplementary.pdf]

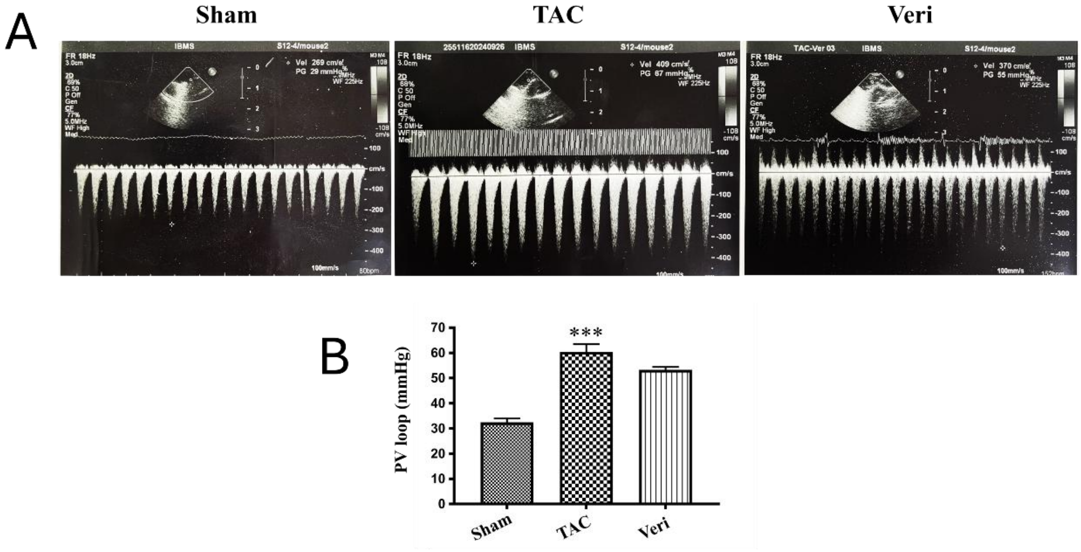


Figure S1: Pressure–Volume Loop Analysis in the Pressure-Overload Model. Representative left ventricular pressure–volume (PV) loops obtained under anesthesia using a 1.2F catheter (Scisense, London, ON, Canada) (A) and quantitative analysis of PV (B) are shown for Sham, transverse aortic constriction (TAC), and TAC + Vericiguat (Veri) groups. TAC significantly increased PV loops compared with Sham (***$p < 0.001$), Data are presented as mean ± SD; $n = 6$ per group. Real-time signals were recorded with LabChart software (ADInstruments, Colorado Springs, CO, USA). Derived indices included end-systolic pressure–volume relationship (ESPVR), preload recruitable stroke work (PRSW), and arterial elastance (Ea), reflecting intrinsic contractility and afterload. All parameters were normalized to body weight and heart rate to minimize inter-animal variability.
